# Cutting vs standard balloon for lesion preparation in drug-coated balloon-only PCI: the NATURE randomised trial

**DOI:** 10.1007/s12928-026-01330-x

**Published:** 2026-08-25

**Authors:** Taku Asano, Masafumi Ono, Norihiro Kogame, Masahiko Noguchi, Mitsuaki Sawano, Raisuke Iijima, Kohei Osakada, Takayuki Ishihara, Koji Nishida, Kenji Ando, Mamoru Nanasato, Takashi Ashikaga, Kengo Tanabe, Takashi Muramatsu, Kiyoshi Hibi, Atsunori Okamura, Yoshihisa Kinoshita, Satoru Suwa, Kozo Okada, Nehiro Kuriyama, Gaku Nakazawa, Masato Nakamura, Satoshi Miyata, Kazushige Kadota, Ken Kozuma

**Affiliations:** 1 Department of Cardiology, St. Luke’s International Hospital, 9-1 Akashi-Cho, Chuo-Ku, P.O. Box 104-8560, Tokyo, Japan; 2 Department of Cardiology, Tokyo Rosai Hospital, Tokyo, Japan; 3 Division of Cardiovascular Medicine, Toho University Ohashi Medical Centre, Tokyo, Japan; 4 Department of Cardiology, Tokyo Bay Urayasu Ichikawa Medical Centre, Chiba, Japan; 5 Teikyo Academic Research Center, Teikyo University, Tokyo, Japan; 6 Department of Cardiovascular Medicine, Kurashiki Central Hospital, Kurashiki, Okayama Japan; 7 Kansai Rosai Hospital Cardiovascular Centre, Hyogo, Japan; 8 Department of Cardiology, Chikamori Hospital, Kochi, Japan; 9 Department of Cardiology, Kokura Memorial Hospital, Fukuoka, Japan; 10 Department of Cardiology, Sakakibara Heart Institute, Tokyo, Japan; 11 Department of Cardiology, Japanese Red Cross Musashino Hospital, Tokyo, Japan; 12 Division of Cardiology, Mitsui Memorial Hospital, Tokyo, Japan; 13 Department of Cardiology, Fujita Health University Hospital, Aichi, Japan; 14 Department of Cardiology, Yokohama City University Hospital, Kanagawa, Japan; 15 Cardiovascular Centre, Sakurabashi Watanabe Advanced Healthcare Hospital, Osaka, Japan; 16 Department of Cardiology, Toyohashi Heart Centre, Toyohashi, Aichi Japan; 17 Department of Cardiology, Juntendo University Shizuoka Hospital, Shizuoka, Japan; 18 Division of Cardiology, Yokohama City University Medical Centre, Kanagawa, Japan; 19 Cardiovascular Centre, Miyazaki Medical Association Hospital, Miyazaki, Japan; 20 Department of Cardiology, Faculty of Medicine, Kindai University, Osaka, Japan; 21 Division of Minimally Invasive Treatment in Cardiovascular Medicine, Toho University Ohashi Medical Centre, Tokyo, Japan; 22 Graduate School of Public Health, Teikyo University School of Medicine, Tokyo, Japan; 23 Division of Cardiology, Department of Internal Medicine, Teikyo University School of Medicine, Tokyo, Japan

**Keywords:** Drug-coated balloon, Cutting balloon, Lesion preparation, De novo coronary artery disease, Fractional flow reserve, Intravascular ultrasound

## Abstract

The success of drug-coated balloon (DCB) therapy for de novo coronary lesions relies on optimal lesion preparation. However, evidence supporting the use of cutting balloons (CBs) for lesion preparation in DCB-only strategies remains limited. The NATURE trial assessed the effectiveness of CB compared with standard balloon (SB) predilatation in achieving optimal lesion preparation for a DCB-only strategy. The NATURE trial was a prospective, multicentre, randomised study conducted at 19 Japanese centres. Patients with de novo coronary lesions suitable for a DCB-only strategy were randomised (1:1) to lesion preparation with CB or SB. Post-predilatation assessment included coronary angiography, intravascular ultrasound (IVUS), and fractional flow reserve (FFR). The primary endpoint was optimal lesion preparation, defined as the simultaneous presence of: (1) no flow-limiting dissection, (2) residual diameter stenosis ≤ 30% by quantitative coronary angiography, and (3) FFR > 0.80. Among 201 patients included in the intention-to-treat population, optimal lesion preparation was achieved in 35.6% of the CB group and 30.0% of the SB group (adjusted risk ratio 1.19, 95% confidence interval 0.87–1.65; p = 0.28). Avoidance of flow-limiting dissection was high in both groups (98.0% vs 97.0%). CB predilatation resulted in lower residual diameter stenosis (30.8 ± 11.8% vs 34.5 ± 13.3%; p = 0.04) and less acute recoil (1.18 ± 0.36 vs 1.31 ± 0.50 mm; p = 0.03). Trends towards larger lumen dimensions on IVUS were also observed with CB. In patients undergoing DCB-only PCI for de novo coronary lesions, CB predilatation provided modest angiographic advantages compared with SB but did not significantly increase the rate of optimal lesion preparation. Optimal lesion preparation is essential for successful drug-coated balloon (DCB) therapy because DCBs do not provide mechanical scaffolding to maintain vessel patency. In this multicentre randomised trial, cutting balloon predilatation resulted in greater acute luminal expansion and less recoil than standard balloon predilatation, although it did not significantly increase the overall rate of optimal lesion preparation defined by angiographic and physiological criteria. These findings suggest that cutting balloons may offer modest mechanical advantages during lesion preparation in DCB-only PCI. However, the clinical significance of these acute angiographic advantages requires confirmation in larger trials and through longer-term follow-up.

**Graphical Abstract:**

**Figure:**
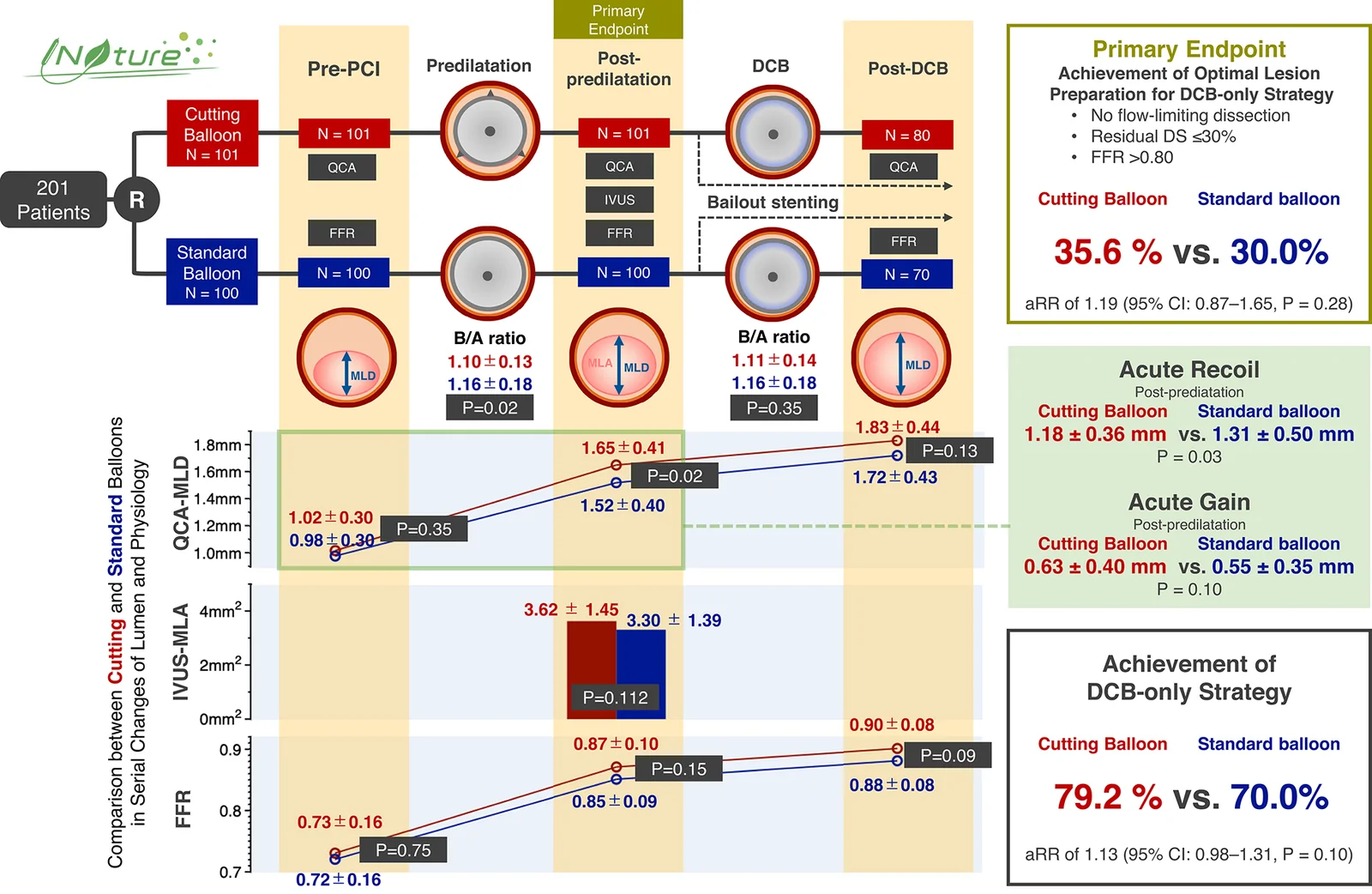

This central illustration summarises the study design, procedural workflow, and key findings of the NATURE trial. Patients with de novo coronary lesions were randomised to lesion preparation with a cutting balloon or a standard balloon before a drug-coated balloon (DCB)–only strategy. Multimodality assessments using quantitative coronary angiography (QCA) and fractional flow reserve (FFR) were performed serially from pre–percutaneous coronary intervention through post-predilatation and post-DCB treatment, and intravascular ultrasound (IVUS) was performed after predilatation. The primary endpoint—achievement of optimal lesion preparation defined as the absence of flow-limiting dissection, residual diameter stenosis ≤ 30%, and FFR > 0.80—was achieved in 35.6% of patients in the cutting balloon group and 30.0% in the standard balloon group. Cutting balloon use was associated with less aggressive predilatation and more favorable acute angiographic outcomes, including greater minimum lumen diameter and lower acute recoil, whereas the overall achievement of optimal lesion preparation and the DCB-only strategy without bailout stenting was comparable between the groups. DCB = drug-coated balloon; FFR = fractional flow reserve; IVUS = intravascular ultrasound; MLD = minimum lumen diameter; MLA = minimum lumen area; PCI = percutaneous coronary intervention; QCA = quantitative coronary angiography.

**Supplementary Information:**

The online version contains supplementary material available at https://doi.org/10.1007/s12928-026-01330-x.

Drug-coated balloons (DCBs) have emerged as a novel alternative to drug-eluting stents (DES) in percutaneous coronary intervention (PCI). This strategy avoids a permanent implant, preserves vasomotion, and may allow for shorter durations of dual antiplatelet therapy [1]. While DCBs were initially proven effective for in-stent restenosis, their application has broadened to de novo small coronary lesions. Clinical trials such as the BASKET-SMALL 2 have demonstrated their safe and efficient use in de novo small coronary lesions [2, 3]. More recently, the use of DCBs for non-small vessels has become a particular area of interest. However, the REC-CAGEFREE I and SELUTION DeNovo trials have shown inconsistent results regarding the non-inferiority of DCBs compared with DES [4–6].

Unlike DES, DCBs provide no mechanical support against elastic recoil. Therefore, the success of a DCB-only strategy for de novo coronary lesions critically depends on optimal lesion preparation to achieve a sufficient and stable lumen, as well as a favorable milieu for effective drug delivery to the vessel wall. The International DCB Consensus Group has proposed criteria for adequate preparation— no flow-limiting dissections and residual diameter stenosis (DS) ≤ 30%—which are now widely referenced [7]. The group also highlighted the potential advantage of specialty balloons, such as cutting balloons (CBs), which employ microblades to create controlled incisions [8]. In addition to controlled incisions, the blades also act as anchoring elements that stabilize the balloon and allow focused plaque modification between blades (pivot or hinge effect). This technique may facilitate vessel expansion at lower pressures and balloon diameters, reduce severe dissections, and create microchannels for deeper drug penetration. This effect has been suggested in a preclinical study in combination with DCB therapy [9].

However, the use of specialty balloons in DCB therapy remains unsubstantiated by robust prospective clinical evidence regarding their acute or long-term efficacy and safety, although their benefit has been established in the treatment of DES in-stent restenosis [10]. To address this gap, this randomised trial was designed as a pilot study to investigate the potential advantages of CBs compared with standard balloons (SBs) for lesion preparation within a DCB-only strategy for de novo coronary lesions.

## Methods

### Study design

The NATURE (Non-stent PCI with an Appropriate Dilatation by Means of Cutting Balloon and Drug-coated Balloon in de novo Lesion) trial is a prospective, multicentre, randomised, open-label, pilot trial. Patients were randomised to either the CB or SB arm for lesion preparation before DCB therapy, to evaluate the rate of successful lesion preparation according to the International DCB Consensus Group criteria. The protocol incorporated intravascular ultrasound (IVUS) and fractional flow reserve (FFR) to provide mechanistic insights into the interaction between lesion preparation and DCB performance.

Blinded core laboratories independently performed analyses of quantitative coronary angiography (QCA) and IVUS, and similarly, a blinded clinical event committee (CEC) adjudicated all clinical events. The trial is registered with the Japan Registry of Clinical Trials (jRCT) system (registration number: JRCTs032230543). The study protocol was approved by the institutional review board or ethics committee at all participating sites, and written informed consent was obtained from all participating patients. The design and rationale of the trial were published elsewhere [11].

Given the paucity of prior data, the trial was intentionally conducted without a formal sample size calculation to generate preliminary evidence for future large-scale studies. Although the present report focuses on findings from the index procedure, further follow-up with angiography will be conducted at nine months, and clinical outcomes data collection at six and 12 months.

### Patient population

The study population consisted of patients with chronic coronary syndrome or stabilised acute coronary syndrome without ongoing troponin elevation, who had de novo coronary lesions deemed suitable for a DCB-only strategy. The key inclusion criteria were patients with 1) a single lesion in a native coronary artery with a visually estimated diameter between 2.5 mm and 4.0 mm on visual estimation, and 2) a lesion length of 28 mm or less. Key exclusion criteria included culprit lesions of acute myocardial infarction (MI), severely calcified lesions requiring atherectomy devices such as rotablator or orbital atherectomy, chronic total occlusions, bifurcation lesions requiring side branch stenting, left main lesions, and ostial lesions. The non-culprit lesions of acute MI are eligible for enrollment if patients are stabilised, following the resolution of the myocardial enzyme peak, and lesions do not have thrombotic characteristics. Detailed inclusion and exclusion criteria are shown in the Supplement Table S1.

### Randomisation and blinding

Eligible patients were randomly assigned in a 1:1 ratio to either the CB group or the SB group using a web-based central registration system (Viedoc™ 4, Viedoc Technologies, Uppsala, Sweden). To minimise imbalances in baseline characteristics between the groups, the Pocock-Simon minimization method was employed, with age (< 75 years vs. ≥ 75 years) and target lesion location (left anterior descending artery vs. non-left anterior descending artery) as stratification factors. Due to the nature of the study, blinding was not possible for either the patient or the operator; however, the core laboratory analysts for QCA and IVUS and the CEC were fully blinded to treatment allocation.

### Study procedure

The operators were asked to adhere to standard PCI procedure techniques while following the protocol described below.

#### Guidewire strategy

To facilitate safe and repeatable FFR measurements during the procedure, particularly for the post-predilatation assessment, the use of a pressure wire as the primary PCI guidewire throughout the procedure was recommended to avoid the risk of exchanging a standard workhorse wire for a pressure wire. Operators were encouraged to use optical pressure wires (COMET™ II, Boston Scientific, Massachusetts, USA) to minimize the risk of signal drift.

#### Lesion preparation (Predilatation)

The Wolverine™ CB (Boston Scientific, Massachusetts, USA) was used in the CB group, while the operator’s choice of a conventional semi-compliant or non-compliant balloon was used in the SB group. Wolverine™ CB employs 127-µm-high microblades that exert highly concentrated mechanical stress to score and fracture atherosclerotic plaque within the target lesion, thereby creating controlled longitudinal micro-incisions [12]. In addition to controlled incisions, the blades also act as anchoring elements that stabilize the balloon and allow focused plaque modification between the blades. A recent finite element analysis corroborates this mechanism, demonstrating that CBs concentrate maximum principal tensile stress precisely at the blade contact points and in the regions between the blades, facilitating effective plaque fracture [12]. Furthermore, this computational modeling revealed that using a CB with a lower B/A ratio (< 1:1) preserves these necessary therapeutic stress levels within the targeted lesion while distinctly reducing stress at the borders of the adjacent normal artery [12].

For balloon sizing, IVUS-based assessment was strongly encouraged over visual estimation from angiography to achieve a balloon-to-artery (B/A) ratio of 1:1, where the artery size referred to the luminal diameter at the healthy segment distal to the lesion.

#### Post-predilatation assessment

As a core component of the study design, the following three assessments were mandatory after completion of lesion preparation and before DCB deployment or bailout stenting: 1) coronary angiography from at least two orthogonal views; 2) IVUS pullback imaging covering the entire treated segment; 3) FFR measurement under pharmacologically induced maximal hyperemia. The position of the pressure sensor during measurement was selected 10–20 mm distal to the target lesion.

#### Decision-making and application of DCB-only strategy

While establishing strict criteria for bailout stenting is generally important in trial designs, definitive multimodality cut-offs for optimal DCB treatment remain unestablished. Therefore, a key secondary objective of this exploratory pilot study was to tune and identify the optimal criteria for DCB therapy utilizing QCA, IVUS, and FFR. Consequently, after the mandatory assessment of the primary endpoint parameters, the final decision to perform bailout stenting was intentionally left to the operator’s discretion rather than being dictated by a rigid protocol. By correlating these acute multimodality parameters and subsequent operator decisions with long-term follow-up data, we anticipate generating robust hypotheses regarding the most appropriate cut-off values for optimal lesion preparation. If the DCB-only strategy was selected, the Agent™ paclitaxel-coated balloon (Boston Scientific, Massachusetts, USA) was used. The DCB size was selected based on a B/A ratio of 1:1 determined by IVUS, and its length was chosen to cover the predilated segment completely. After DCB deployment, a waiting period of 5 to 20 min was recommended to confirm the absence of acute elastic recoil or vessel occlusion. The Synergy™ everolimus-eluting stent (Boston Scientific, Massachusetts, USA) was used in the case of bailout stenting. Final angiography and a final IVUS pullback were performed after DCB deployment or stent placement.

### Angiography analysis

Off-line QCA analysis was performed by an independent core laboratory (CARDIOCORE Japan, Tokyo, Japan) using the QAngioXA system (Medis Medical Imaging, Leiden, the Netherlands) according to standard protocols [13]. Analysis was performed at each time point of pre-PCI, post-final predilatation, and post-PCI. Dissections were classified using the National Heart, Lung, and Blood Institute criteria (type A through F) [14]. The B/A ratio was calculated by dividing the nominal diameter of the applied balloon by the baseline reference vessel diameter.

### IVUS imaging analysis

Off-line IVUS analysis was performed on the images acquired after the final predilatation by an independent core laboratory (St. Luke’s International Hospital, Tokyo, Japan) using QIvus (Medis Medical Imaging, Leiden, the Netherlands), including lumen and external elastic membrane (EEM) areas according to standard protocols [15, 16]. All pullbacks were analysed at 1-mm longitudinal intervals. The reference segments were defined as the 5-mm segments proximal and distal to the site where the device (DCB or stent) was deployed. Because the exact implant location of the DCB was unclear, the segment was defined based on anatomical landmarks such as side branches and calcification by referring to cine angiography. The cross-sectional lumen border was delineated to include dissection cavities in which no blood accumulation was observed. The maximum cross-sectional dissection arc, the longitudinal length of the dissection, and the dissection depth were measured. The dissection depth was classified as follows: intimal, medial, adventitial, and intramural hematoma [15].

### Endpoints and definitions

#### Primary endpoint

The primary endpoint of the study was the success rate of optimal lesion preparation, as assessed by angiographic parameters analysed in an independent core laboratory and by FFR values obtained after predilatation. This composite endpoint was defined as “successful” if all three of the following criteria were met simultaneously after predilatation: (1) absence of flow-limiting dissection resulting in a Thrombolysis in Myocardial Infarction (TIMI) flow grade < 3, (2) residual DS ≤ 30%, and (3) FFR > 0.80.

At the time of the protocol design, the criteria for optimal lesion preparation were defined based on the third report of the International DCB Consensus Group, which represented the mainstream procedural targets by recommending a residual diameter stenosis (DS) of ≤ 30% and a fractional flow reserve (FFR) of > 0.80 [7]. However, as recently highlighted by the Drug-Coated Balloon Academic Research Consortium (DCB-ARC) position statement, definitive evidence-based thresholds for optimal residual DS and physiological indices such as FFR remain unestablished [17]. Recognizing this evolving perspective, a secondary objective of this study is to conduct post-hoc analyses to investigate the clinical validity and prognostic relevance of these specific cut-off values by correlating the acute angiographic and physiological results with long-term clinical and angiographic follow-up data.

#### Procedural outcomes and 30-day clinical endpoints

The operator’s decision to employ a DCB-only strategy after predilatation, as well as the subsequent success rate of this strategy without the need for bailout stenting, was evaluated in the two groups. The 30-day clinical endpoints included all-cause death, cardiovascular death, MI—including periprocedural MI defined according to the Society for Cardiovascular Angiography and Interventions (SCAI) criteria—clinically indicated target lesion revascularization (TLR), abrupt vessel occlusion, and stent thrombosis [18].

#### Sample size calculation

As a pilot study, the sample size (N = 200) was chosen to generate foundational data in the absence of similar studies and was not based on a formal power calculation for clinical endpoints. The target sample size was set as a feasible number of participants that could be enrolled within the study period, with no assumptions made regarding superiority or non-inferiority.

#### Statistical analysis

The primary analysis was performed in the intention-to-treat (ITT) population. A per-protocol set (PPS) analysis was conducted as a sensitivity analysis for the primary endpoint. The PPS was defined as the population excluding patients who lacked QCA or FFR data for assessment of the primary endpoint and those who underwent crossover treatment.

Categorical variables were compared between groups using Fisher’s exact test, and continuous variables were compared using Welch’s t-test, as appropriate. Adjusted analyses estimating risk ratios were conducted using modified Poisson regression with robust standard errors, adjusting for age and lesion location—the variables used to balance randomisation—and including enrollment site as a mixed effect. Results are presented as adjusted risk ratios (aRRs) with corresponding 95% confidence intervals (CIs) to illustrate the magnitude and precision of the observed effects for each endpoint in the ITT and PPS analyses.

All statistical analyses were performed using SAS software (version 9.4; SAS Institute, Cary, NC, USA). Statistical significance was defined as a two-sided p-value < 0.05; however, p-values were interpreted in the context of an exploratory, hypothesis-generating analysis rather than as definitive evidence of treatment differences.

## Results

### Patient and lesion characteristics

**Fig. 1:**
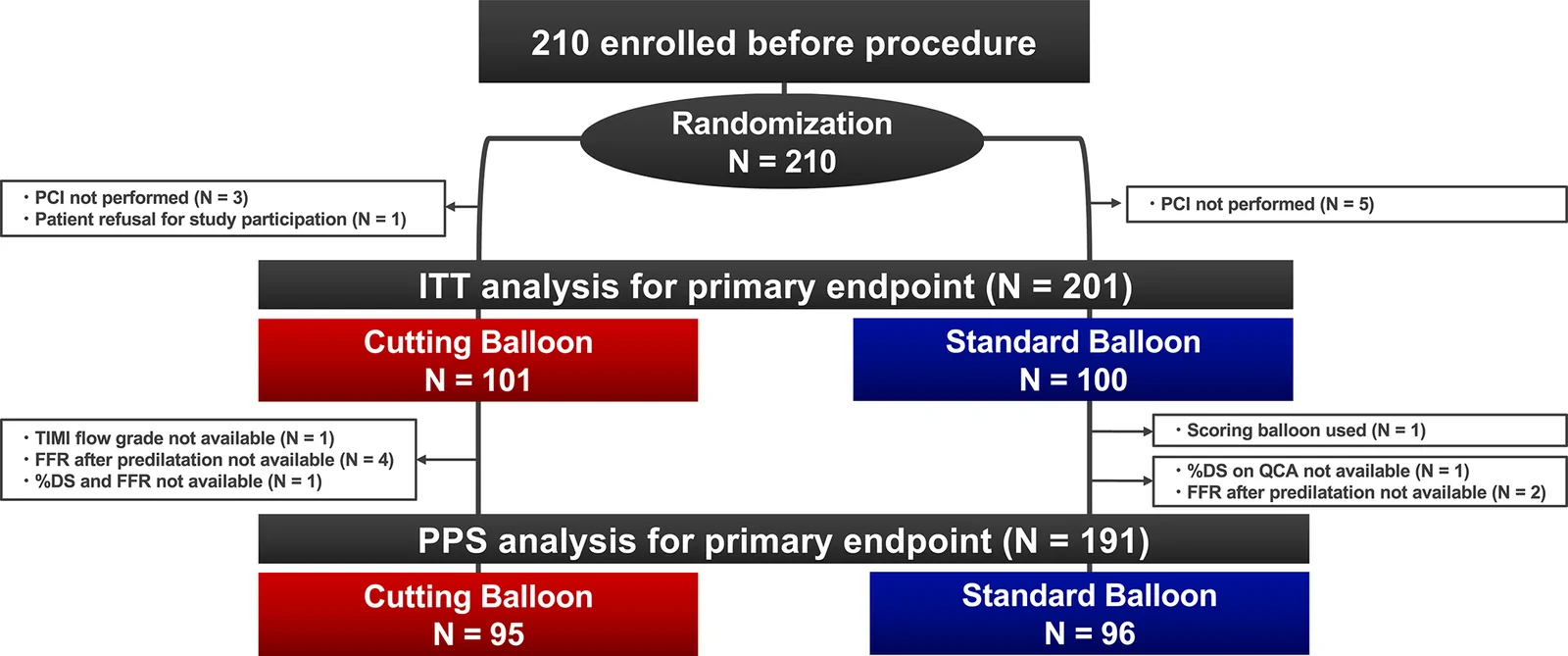
Study flow chart. A total of 210 patients were enrolled and randomised before the index procedure. After exclusion of patients who did not undergo PCI or withdrew consent, 201 patients were included in the intention-to-treat (ITT) population for the primary endpoint analysis (Cutting Balloon group, n = 101; Standard Balloon group, n = 100). After exclusion of protocol deviations and missing core laboratory–assessed data, 191 patients were included in the per-protocol set (PPS) analysis (Cutting Balloon group, n = 95; Standard Balloon group, n = 96). Reasons for exclusion from each analysis are shown. DS = diameter stenosis; FFR = fractional flow reserve; ITT = intention-to-treat; PCI = percutaneous coronary intervention; PPS = per-protocol set; QCA = quantitative coronary angiography; TIMI = Thrombolysis in Myocardial Infarction

Between February 2024 and May 2025, a total of 210 patients were enrolled and randomised at 19 Japanese sites listed in Supplement Table S2. Among them, eight patients did not undergo PCI because the operators deemed the procedure unnecessary after confirming a pre-procedural FFR value > 0.80, and one patient withdrew consent before PCI. A total of 201 patients were included in the ITT population, with 101 assigned to the CB group and 100 to the SB group (Fig. 1). In the ITT population, one patient in the CB group was excluded from the IVUS analysis because no post-predilatation IVUS images were recorded, although IVUS-guided PCI had been performed. Baseline characteristics were comparable between the two groups (Table 1); mean age was similar (70.0 vs. 70.9 years), as was the proportion of female patients (22.8% vs. 20.0%), and the prevalence of diabetes mellitus (36.6% vs. 34.0%) did not differ substantially between groups. Procedural characteristics are shown in Table 2. Compared with SB, CB was associated with less aggressive predilatation, characterised by a lower maximum inflation pressure (9.90 ± 3.28 vs. 14.31 ± 4.45 atm, P < 0.001) and a lower B/A ratio (1.10 ± 0.13 vs. 1.16 ± 0.18, P = 0.02), whereas maximum balloon diameter did not differ significantly between the two groups. In the SB group (N = 100), lesion preparation was ultimately performed using non-compliant balloons in 65 patients, semi-compliant balloons in 31 patients, cutting balloons in three patients, and a scoring balloon in one patient. The frequent use of non-compliant balloons reflects an intention to avoid excessive vessel stretch during dilatation, although the choice between semi-compliant and non-compliant balloons was also influenced by institutional preferences. In the three cases where cutting balloons were used, the operators initially utilized an SB but required subsequent CB dilatation, according to the study protocol, to achieve an adequate lumen to safely complete the PCI procedure. These cases were included in the PPS analysis, whereas the one case involving upfront use of a scoring balloon was excluded as a protocol deviation.

**Table 1:** Baseline characteristics

	Cutting balloon N = 101	Standard balloon N = 100
Age, years	70.0 ± 10.9	70.9 ± 11.1
Sex, female	23 (22.8%)	20 (20.0%)
BMI	24.4 ± 3.4	25.1 ± 3.8
Dyslipidemia	72 (71.3%)	83 (83.0%)
Hypertension	82 (81.2%)	83 (83.0%)
Diabetes mellitus	37 (36.6%)	34 (34.0%)
Peripheral arterial disease	6 (5.9%)	5 (5.0%)
Current smoker	15 (14.9%)	16 (16.0%)
Chronic renal insufficiency	14 (13.9%)	8 (8.0%)
LVEF, %	59.2 ± 10.8	57.9 ± 10.9
Prior PCI	44 (43.6%)	46 (46.0%)
Prior CABG	0 (0%)	0 (0%)
Prior MI	22 (21.8%)	24 (24.0%)
Prior stroke	7 (6.9%)	7 (7.0%)
Family history of CAD	14 (13.9%)	8 (8.0%)
Stable angina	70 (69.3%)	64 (64.0%)
Silent myocardial ischemia	18 (17.8%)	15 (15.0%)
Unstable angina	1 (1.0%)	9 (9.0%)
Stabilized MI (PCI for non-culprit lesion)	12 (11.9%)	12 (12.0%)

BMI = body mass index; CABG = coronary artery bypass grafting; CAD = coronary artery disease; LVEF = left ventricular ejection fraction; MI = myocardial infarction; PCI = percutaneous coronary intervention.

**Table 2:** Lesion and procedural characteristics

	Cutting balloon N = 101	Standard balloon N = 100	P value
**Lesion characteristics**			
Number of lesions	101	100	
Lesion location			0.92
RCA	30 (29.7%)	31 (31.0%)	
LAD	41 (40.6%)	42 (42.0%)	
LCX	30 (29.7%)	27 (27.0%)	
AHA classification			0.24
Type A	19 (18.8%)	15 (15.0%)	
Type B1	36 (35.6%)	48 (48.0%)	
Type B2	42 (41.6%)	31 (31.0%)	
Type C	4 (4.0%)	6 (6.0%)	
Lesion length			0.54
Discrete	46 (45.5%)	39 (39.0%)	
Tubular	52 (51.5%)	56 (56.0%)	
Diffuse	3 (3.0%)	5 (5.0%)	
Lesion morphology			
Eccentric	35 (34.7%)	31 (31.0%)	0.65
Ostial lesion	2 (2.0%)	0 (0.0%)	0.50
Calcification			0.43
None/mild calcification	92 (91.1%)	87 (87.0%)	
Moderate calcification	9 (8.9%)	12 (12.0%)	
Severe calcification	0 (0.0%)	1 (1.0%)	
Bifurcated lesion	19 (17.8%)	16 (16.0%)	0.92
**Procedure characteristics**			
	**N = 101**	**N = 100**	
Procedure time	73.3 (37.6)	73.2 (32.7)	0.98
**Predilatation**	**N = 101**	**N = 100**	
Maximum balloon diameter, mm	2.83 ± 0.50	2.84 ± 0.55	0.94
Maximum balloon length, mm	10.73 ± 2.66	14.46 ± 2.95	< 0.001
Maximum inflation pressure, atm	9.90 ± 3.28	14.31 ± 4.45	< 0.001
Balloon-to-artery ratio	1.10 ± 0.13	1.16 ± 0.18	0.02
**DCB therapy**	**N = 83**	**N = 72**	
Maximum balloon diameter, mm	2.87 ± 0.55	2.88 ± 0.57	0.90
Maximum balloon length, mm	19.94 ± 5.49	22.64 ± 7.69	0.01
Maximum inflation pressure, atm	7.46 ± 2.19	7.24 ± 2.70	0.58
Inflation time, sec	56.75 ± 14.89	51.46 ± 16.02	0.04
Balloon-to-artery ratio	1.11 ± 0.14	1.16 ± 0.18	0.05
**Bailout stenting**	**N = 21**	**N = 30**	
Maximum stent diameter, mm	3.02 ± 0.47	2.93 ± 0.54	0.49
Maximum stent length, mm	24.43 ± 10.47	27.13 ± 11.92	0.40
Maximum inflation pressure, atm	12.33 ± 2.2	12.30 ± 2.82	0.96

AHA = American Heart Association; DCB = drug-coated balloon; LAD = left anterior descending artery; LCX = left circumflex artery; RCA = right coronary artery.

### Primary endpoint analysis

**Fig. 2:**
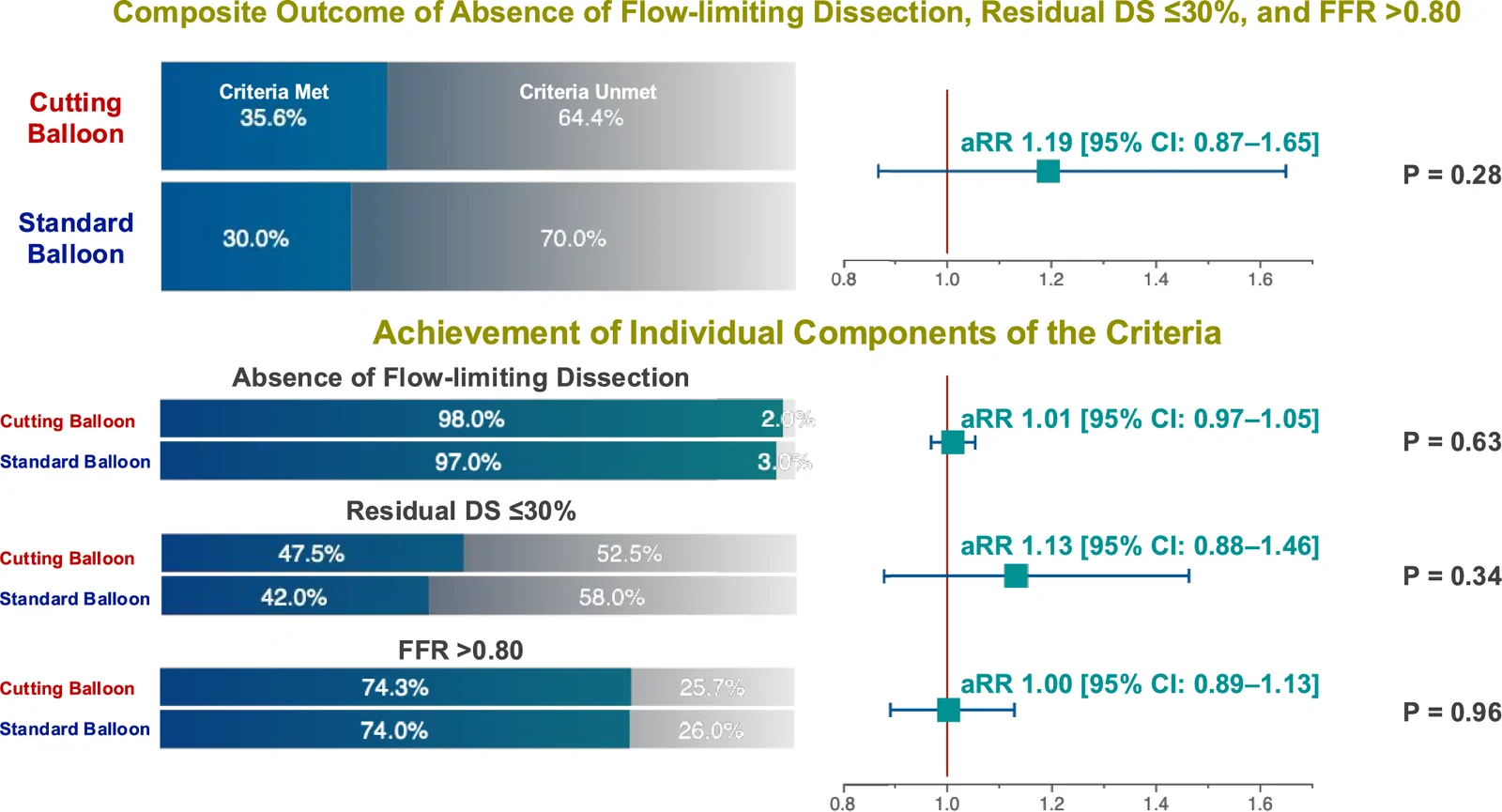
Primary endpoint: achievement of optimal lesion preparation. The composite primary endpoint—defined as the absence of flow-limiting dissection, residual diameter stenosis (DS) ≤ 30% by quantitative coronary angiography, and fractional flow reserve (FFR) > 0.80 after predilatation—was achieved in 35.6% of patients in the cutting balloon group and 30.0% in the standard balloon group. The forest plot shows adjusted relative risks (aRRs) with 95% confidence intervals for the composite endpoint and for each individual component. No significant differences were observed between the groups for the composite endpoint or any individual component. aRR = adjusted relative risk; DS = diameter stenosis; FFR = fractional flow reserve

The composite primary endpoint—successful optimal lesion preparation—was achieved in 35.6% (36/101 patients) of the CB group compared to 30.0% (30/100 patients) in the SB group, corresponding to an aRR of 1.19 (95% CI: 0.87–1.65, P = 0.28) (Fig. 2). When analysing the individual components of the primary endpoint, the rate of absence of flow-limiting dissection was 98.0% in the CB group and 97.0% in the SB group, with aRR of 1.01 (95% CI: 0.97–1.05, P = 0.63). The achievement rate of residual DS ≤ 30% by QCA was 47.5% in the CB group and 42.0% in the SB group, with aRR of 1.13 (95% CI: 0.88–1.46, P = 0.34). The achievement rate of FFR > 0.80 was 74.3% in the CB group and 74.0% in the SB group, with aRR of 1.00 (95% CI: 0.89–1.13, P = 0.96). In the PPS analysis, the primary endpoint results were consistent and are presented in the Supplement Table S3.

### Anatomical and physiological outcomes after predilatation

The detailed QCA at three phases (pre-PCI, post-predilatation, and post-PCI) and post-predilatation IVUS and FFR results are presented in Table 3. The CB group demonstrated significantly higher post-predilatation minimum lumen diameter (MLD) (1.65 ± 0.41 vs. 1.52 ± 0.40 mm; P = 0.02), significantly lower post-predilatation acute recoil (1.18 ± 0.36 vs. 1.31 ± 0.50 mm; P = 0.03); furthermore, numerically higher post-predilatation acute gain (0.63 ± 0.40 vs. 0.55 ± 0.35 mm; P = 0.10). The CB group demonstrated significantly lower residual DS (30.8 ± 11.8 vs. 34.5 ± 13.3%; P = 0.04).

**Table 3:** Results of QCA, IVUS, and FFR analyses

	Cutting balloon N = 101	Standard balloon N = 100	P value
**CA**			
**Pre-predilatation**	**N = 101**	**N = 100**	
Minimum lumen diameter, mm	1.02 ± 0.30	0.98 ± 0.30	0.35
Diameter stenosis, %	60.5 ± 10.1	60.6 ± 9.7	0.91
Reference vessel diameter, mm	2.60 ± 0.55	2.50 ± 0.55	0.17
Lesion length, mm	11.70 ± 4.87	11.52 ± 4.32	0.79
**Post-predilatation**	**N = 100**	**N = 99**	
Minimum lumen diameter, mm	1.65 ± 0.41	1.52 ± 0.40	0.02
Diameter stenosis, %	30.8 ± 11.8	34.5 ± 13.3	0.04
Acute gain, mm	0.63 ± 0.40	0.55 ± 0.35	0.10
Acute recoil, mm	1.18 ± 0.36	1.31 ± 0.50	0.03
**Post-DCB-only strategy**	**N = 80**	**N = 70**	
Minimum lumen diameter, mm	1.83 ± 0.44	1.72 ± 0.43	0.13
Diameter stenosis, %	26.7 ± 11.3	29.4 ± 11.4	0.15
Acute gain, mm	0.80 ± 0.42	0.73 ± 0.37	0.26
Acute recoil, mm	1.05 ± 0.39	1.16 ± 0.39	0.08
**Post-bailout stenting**	**N = 21**	**N = 30**	
Minimum lumen diameter, mm	2.57 ± 0.32	2.45 ± 0.46	0.30
Diameter stenosis, %	12.5 ± 5.6	13.6 ± 5.6	0.49
Acute gain, mm	1.57 ± 0.31	1.49 ± 0.42	0.45
Acute recoil, mm	0.46 ± 0.34	0.47 ± 0.33	0.87
**IVUS**			
**Post-predilatation**	**N = 100**	**N = 100**	
Proximal segment			
Mean lumen area, mm^2^	8.87 ± 4.92	8.16 ± 3.72	0.25
Mean EEM area, mm^2^	15.87 ± 6.37	15.24 ± 5.91	0.47
Target segment			
Minimum lumen area, mm^2^	3.62 ± 1.45	3.30 ± 1.39	0.11
Minimum EEM area, mm^2^	9.84 ± 4.02	9.44 ± 4.59	0.51
Maximum plaque burden, %	70.75 ± 6.19	72.25 ± 6.58	0.10
Area stenosis, %	52.18 ± 12.60	53.62 ± 12.97	0.43
Maximum plaque area, mm^2^	10.43 ± 3.64	10.60 ± 4.87	0.78
Minimum eccentricity index	0.60 ± 0.10	0.60 ± 0.10	0.72
Maximum arc of dissection, degree	88.9 ± 53.9	99.7 ± 60.7	0.18
Maximum length of dissection, mm	10.6 ± 8.5	9.8 ± 7.4	0.46
Distal segment			
Mean lumen area, mm^2^	7.02 ± 3.32	6.68 ± 3.38	0.48
Mean EEM area, mm^2^	11.47 ± 4.98	11.45 ± 5.85	0.98
**FFR**			
**Pre-predilatation**	**N = 100**	**N = 97**	
FFR	0.73 ± 0.16	0.72 ± 0.16	0.75
**Post-predilatation**	**N = 96**	**N = 98**	
FFR	0.87 ± 0.10	0.85 ± 0.09	0.15
**Post-DCB-only strategy**	**N = 77**	**N = 67**	
FFR	0.90 ± 0.08	0.88 ± 0.08	0.09
**Post-bailout stenting**	**N = 19**	**N = 28**	
FFR	0.89 ± 0.08	0.87 ± 0.07	0.44

DCB = drug-coated balloon; EEM = external elastic membrane; FFR = fractional flow reserve; IVUS = intravascular ultrasound; MLA = minimum lumen area; MLD = minimum lumen diameter; QCA = quantitative coronary angiography.

In the IVUS analysis, the CB group had a numerically greater post-predilatation MLD (2.11 ± 0.41 vs. 2.01 ± 0.43 mm; P = 0.09) and minimum lumen area (MLA) (3.62 ± 1.45 mm^2^ vs. 3.30 ± 1.39 mm^2^; P = 0.11); consistent with the QCA results. Post-predilatation FFR values were numerically higher in the CB group (0.87 ± 0.10 vs. 0.85 ± 0.09; P = 0.15).

**Fig. 3:**
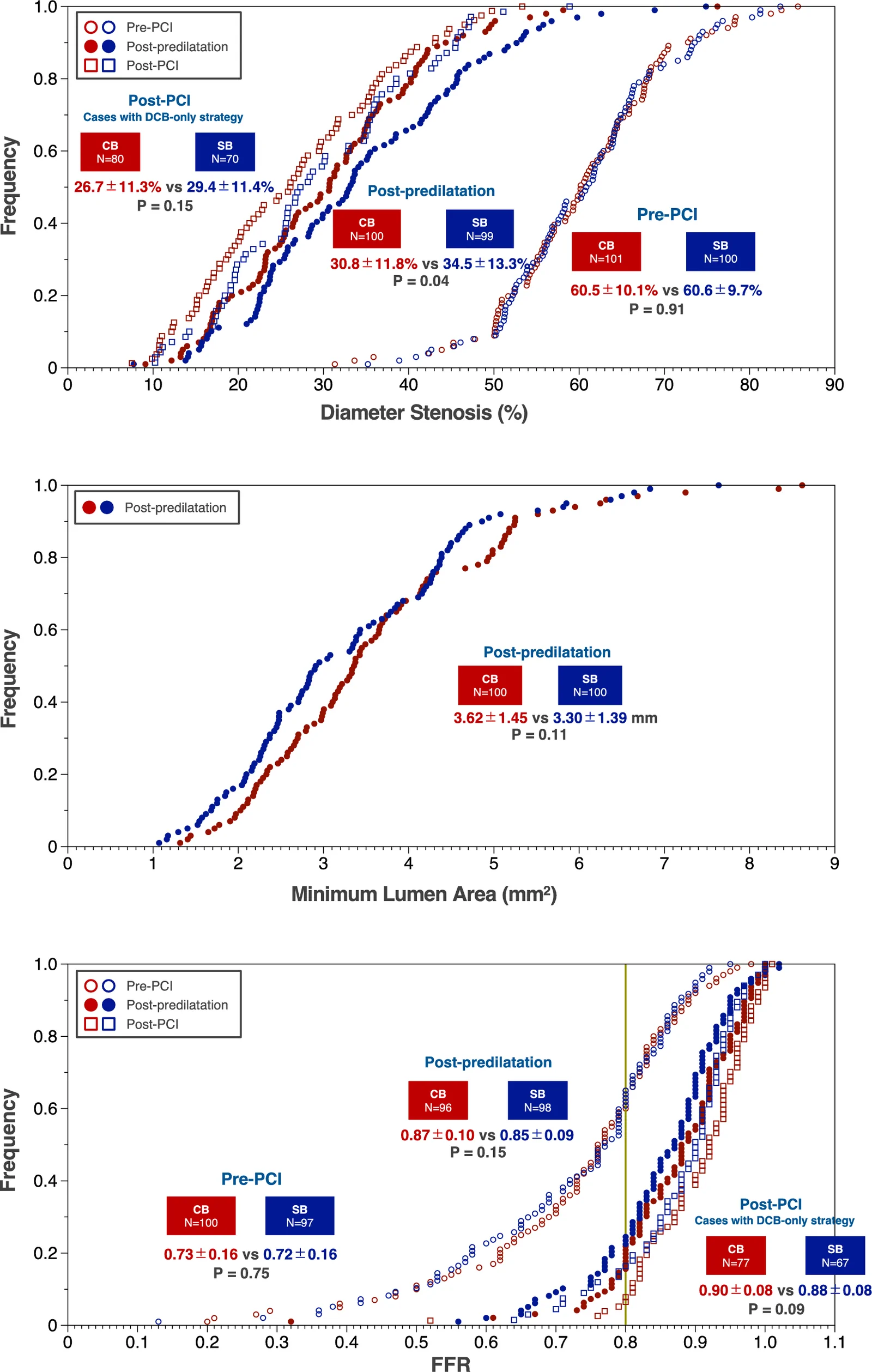
Cumulative frequency distributions of post-predilatation Diameter Stenosis on Angiography, Minimum Lumen Area on IVUS, and FFR. Cumulative frequency distributions of post–predilatation diameter stenosis assessed by quantitative coronary angiography, minimum lumen area assessed by intravascular ultrasound, and fractional flow reserve are shown for the cutting balloon and standard balloon groups. FFR = fractional flow reserve; IVUS = intravascular ultrasound

**Fig. 4:**
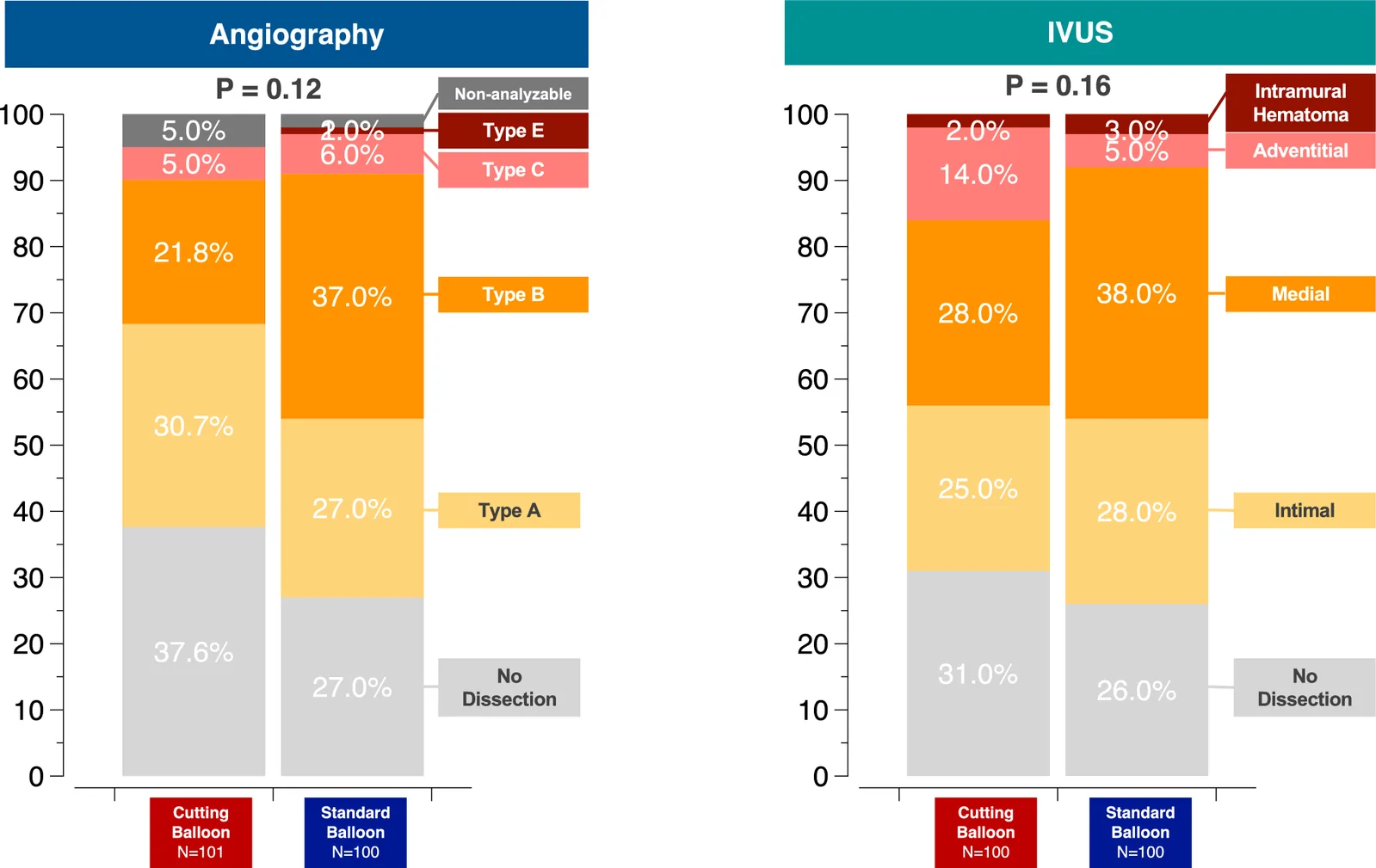
Dissection assessment post-predilatation on Angiography and IVUS. The distribution of coronary dissections after predilatation assessed by angiography (left panel) and intravascular ultrasound (right panel) is shown for the cutting balloon and standard balloon groups. Angiographic dissections were classified according to the National Heart, Lung, and Blood Institute criteria, whereas IVUS-detected dissections were categorised by vessel wall layer involvement. No significant differences were observed between the groups in the overall distribution of dissection severity by either imaging modality. FFR = fractional flow reserve; IVUS = intravascular ultrasound

Figure 3 illustrates the cumulative frequency distribution curves for the two groups, specifically DS on QCA, MLA on IVUS, and FFR, respectively. The incidence and classification of dissection assessed by angiography and IVUS, as well as the arc and length of dissection analysed on IVUS, were comparable between the two groups (Table 3 and Fig. 4).

### Procedural and 30-day clinical outcomes

**Fig. 5:**
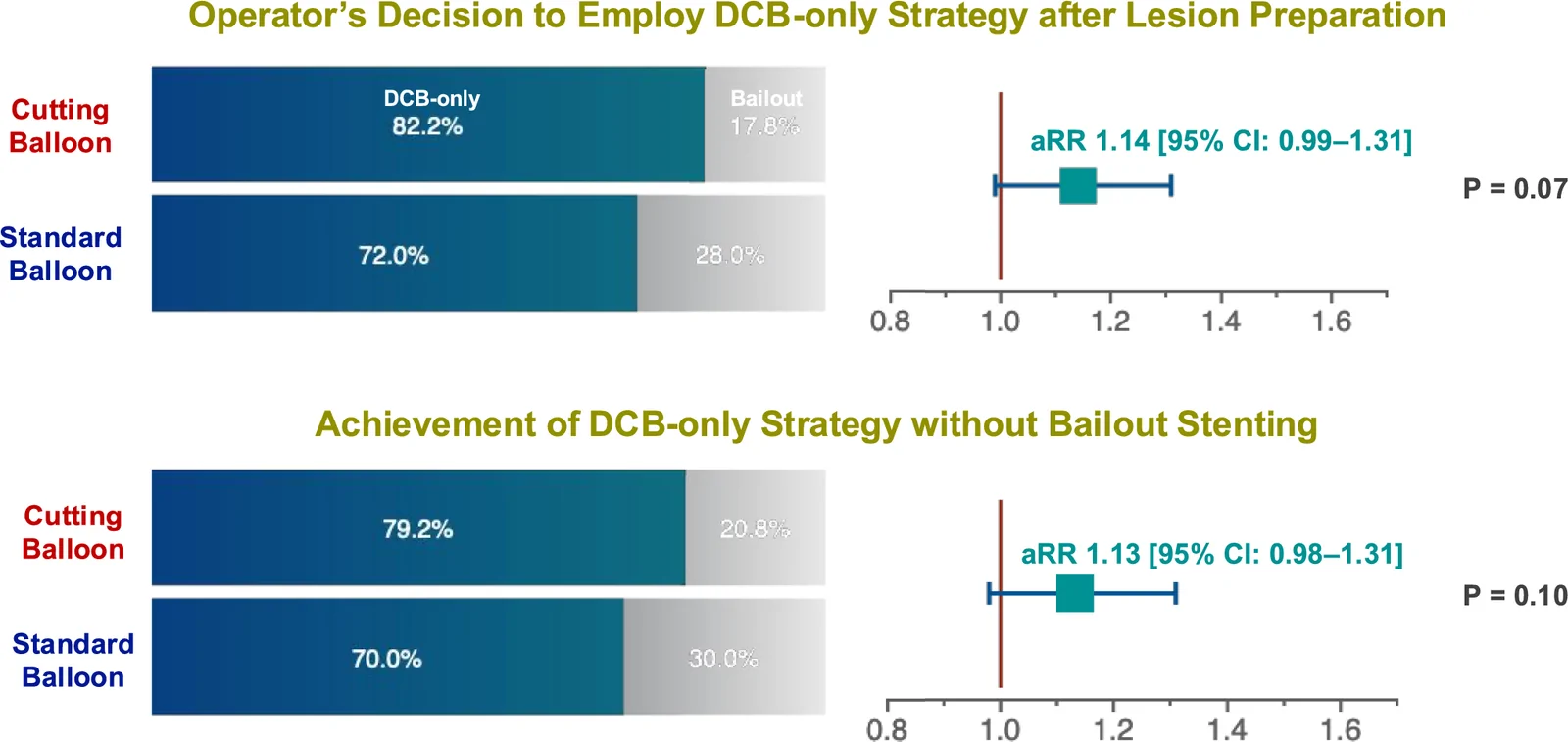
Operator’s decision and achievement of DCB-only strategy. The upper panel shows the operator’s decision to employ a drug-coated balloon (DCB)–only strategy after lesion preparation in the cutting balloon and standard balloon groups. The lower panel shows the achievement of the DCB-only strategy without bailout stenting. Bars represent the proportion of patients managed with a DCB-only strategy versus bailout stenting. Forest plots display adjusted relative risks (aRRs) with 95% confidence intervals for each comparison. aRR = adjusted relative risk; DCB = drug-coated balloon

The operator’s decision to employ a DCB-only strategy after predilatation was made in 82.2% (83/101) for the CB group and 72.0% (72/100) for the SB group with an aRR of 1.14 (95%CI: 0.99–1.31, P = 0.07) (Fig. 5). Of those patients, three patients in the CB group and two patients in the SB group received bailout stenting after DCB deployment. Finally, the achievement rates of the DCB-only strategy were 79.2% (80/101) in the CB group and 70.0% (70/100) in the SB group, respectively, with an aRR of 1.13 (95%CI: 0.98–1.31, P = 0.10).

In both allocation groups, patients who required bailout stenting tended to exhibit smaller luminal dimensions (e.g., MLD and MLA), greater residual DS, more severe angiographic dissections, deeper dissections on IVUS, and lower FFR values after predilatation compared with those successfully treated with a DCB-only strategy (Supplemental Table 4).

In the PPS analysis, the operator’s decision to employ a DCB-only strategy was significantly more frequent in the CB group than in the SB group (85.3% vs. 71.9%), corresponding to an aRR of 1.19 (95% CI: 1.02–1.39; P = 0.03). The achievement rates of the DCB-only strategy were 82.1% and 69.8% in the CB and SB groups, respectively, corresponding to an aRR of 1.18 (95% CI: 1.00–1.38; P = 0.04) (Supplement Table 5).

During the 30-day follow-up period, there were no occurrences of death or clinically indicated TLR in either group. The incidence of all MI was 2.0% (2/101) in the CB group and 4.0% (4/100) in the SB group, with an aRR of 0.50 (95%CI: 0.11–2.33, P = 0.38). All MIs were periprocedural, and no cases of abrupt vessel occlusion or stent thrombosis were observed.

## Discussion

The NATURE trial is the first prospective, randomized controlled trial to compare CB and SB predilatation for lesion preparation in a DCB–only strategy for de novo coronary lesions using comprehensive multimodality assessments, including core laboratory–analysed QCA, IVUS, and FFR. The primary endpoint, defined as achievement of optimal lesion preparation according to prespecified angiographic and physiological criteria, was comparable between the two groups. Although CB predilatation resulted in modest angiographic advantages such as greater minimum lumen diameter, lower residual diameter stenosis, and less acute recoil, these differences did not translate into a significantly higher rate of optimal lesion preparation (Central Illustration).

### The achievement rate of optimal lesion preparation criteria

High avoidance rates of flow-limiting dissections were achieved by both groups (98.0% and 97.0%), indicating that SB also allowed for safe treatment without inducing malignant dissections, and that the dissection-controlling design of CB did not translate into a measurable advantage. This comparable safety may have been influenced by the use of IVUS to guide DCB preparation. In line with the difference in residual DS values, the achievement of residual DS ≤ 30% tended to be higher in the CB group; however, the wide confidence intervals suggest that the trial lacked sufficient power to demonstrate a definitive benefit.

Regarding the physiological assessment, we adopted a post-predilatation FFR > 0.80 as a criterion for optimal lesion preparation based on the Third Report of the International DCB Consensus Group published in 2020. At the time of our protocol design, the consensus document suggested that an FFR of 0.80 might serve as a "good compromise" to guide DCB angioplasty, bridging historical targets of > 0.90 and more recent cutoffs of 0.85 or 0.75. However, as noted in the recently published DCB-ARC position statement, a specific physiological threshold cannot be definitively recommended at this time due to the limited and preliminary nature of available data on coronary physiology during DCB treatment. Therefore, the physiological findings in the current acute phase remain hypothesis-generating. A crucial exploratory goal of our trial is to further scrutinize the validity and clinical relevance of this 0.80 threshold by comprehensively correlating the acute physiological results with long-term angiographic and clinical outcomes.

Despite the trend toward higher post-predilatation FFR values in the CB group, the achievement rate of FFR > 0.80 was virtually identical between the two groups. The luminal gain achieved by CB may therefore have had a limited functional impact. Interestingly, post-PCI FFR values did not differ between patients treated with a DCB-only strategy and those who underwent bailout stenting, although residual DS after bailout stenting was more favourable than that after the DCB-only strategy (Table 3). One possible explanation is that additional stenting provides limited incremental improvement in coronary flow once adequate lumen expansion has already been achieved with balloon dilatation. An early physiological study using Doppler flow measurements demonstrated that although stent implantation after balloon angioplasty further improved angiographic lumen dimensions, the corresponding increase in coronary flow velocity was modest [19]. These findings suggest that coronary flow may reach a physiological plateau after effective balloon dilatation. The luminal differences between CB and SB, therefore, likely reflect differences in barotrauma rather than functional significance, and their implications for long-term outcomes in DCB-only strategies warrant further investigation.

### The low achievement rate of optimal lesion preparation criteria

Notably, fewer cases than expected achieved the fulfilment of the optimal lesion preparation criteria. This was presumably attributable to the low residual DS ≤ 30% in this DCB-only strategy (47.5% vs. 42.0%). Although the International DCB Consensus Group recommends DS ≤ 30% based on visual estimation, which inherently differs from QCA-derived measurements, the operators often proceeded with a DCB-only approach despite leaving greater residual stenosis than anticipated. Visual estimation of DS is known to have low reproducibility and to be highly susceptible to bias from various factors, including clinical information [13, 20, 21]. Pre-PCI visually estimated DS tends to be overestimated relative to QCA-DS. Conversely, post-predilatation DS may be subconsciously underestimated to justify proceeding without stent implantation, as suggested by the observed results showing higher residual DS values despite the prespecified residual DS criteria.

From a functional perspective, a subanalysis of the FAME study demonstrated that many lesions with visually estimated 50–70% diameter stenosis had an FFR > 0.80, and that even among lesions with 71–90% stenosis, a notable proportion remained functionally non-significant [22]. These findings highlight a mismatch between visual DS categories and the FFR-based cutoff for ischemia. A similar pattern was observed in our study, particularly in lesions of the right coronary and left circumflex arteries, where many lesions exhibited an FFR > 0.80 despite residual DS > 30% (Supplement Figure S1).

Consequently, the observed low achievement rate of the primary composite endpoint, which was primarily driven by the stringent predefined criteria, particularly the strict thresholds for residual diameter stenosis. Notably, even among lesions that did not meet the primary endpoint criteria for optimal lesion preparation, a DCB-only strategy was ultimately achieved in a substantial proportion of patients, specifically in 70.8% (46/65) of the CB group and 61.4% (43/70) of the SB group. This indicates that operators frequently proceeded with a DCB-only approach at their discretion despite the presence of suboptimal angiographic or physiological findings based on the stringent study criteria. The fact that patients with smaller luminal dimensions, greater residual stenosis, or lower FFR values were more likely to receive bailout stenting indicates that these multimodality parameters were crucial in the operators’ intra-procedural decision-making (Supplement Table 4). However, the exact cut-offs defining optimal DCB preparedness remain to be revisited. As supported by the recent DCB-ARC position statement, definitive evidence-based cut-offs for angiographic and physiological parameters remain unestablished, suggesting that the criteria utilized in our trial may have been unduly high for real-world practice. Furthermore, acute morphological and functional assessments alone may not fully reflect the subsequent biological healing processes unique to DCB therapy, such as late lumen enlargement. Therefore, it is imperative to correlate these acute procedural metrics with our upcoming 9-month angiographic and 1-year clinical follow-up data. By integrating these long-term outcomes, future analyses will aim to explore and validate more appropriate, clinically relevant criteria for optimal lesion preparation, ultimately refining the procedural standards and decision-making process for DCB-only strategies.

### The potential acute advantage of CB in the DCB-only strategy: insights from this trial

The design of a CB—specifically, its ability to create intentional incisions—may contribute to achieving better acute results regarding luminal gain. The acquired larger lumen after lesion preparation with CBs may be a crucial decision factor for the DCB-only strategy, avoiding bailout stenting. In this context, the DCB-only strategy was applied in a larger proportion of patients in the CB group compared with the SB group, although these operators’ decisions were made in an unblinded manner (Fig. 4). Preclinical histological evaluations have demonstrated greater disruption of the internal elastic lamina (IEL) with CB compared with SB [9]. This finding suggests that the superior acute lumen enlargement and reduced acute recoil observed after CB predilatation may result from effective IEL disruption. However, these experimental mechanisms may not fully extrapolate to complex atherosclerotic lesions in humans [9].

It should be noted that while CB predilatation demonstrated angiographic advantages, such as lower residual diameter stenosis and reduced acute recoil on QCA, these improvements were not accompanied by statistically significant differences in IVUS parameters. This discrepancy between the imaging modalities may be explained by their inherent technical differences. While QCA evaluates the two-dimensional diameter, IVUS measures the true cross-sectional area. QCA generally tends to underestimate the actual lumen size compared to IVUS [23]. Furthermore, alterations in the luminal surface following balloon dilatation can disturb the laminar flow of the contrast media, potentially rendering QCA less accurate than IVUS in assessing the true post-dilatation lumen. Physiologically, coronary vascular function and flow correlate more closely with cross-sectional area than with diameter. In our trial, the lack of significant difference in IVUS-derived area is consistent with the absence of a significant difference in post-predilatation FFR, a functional index reflecting coronary flow. Consequently, whether the acute angiographic advantages observed in the CB group truly reflect superior lesion expansion and are clinically meaningful remains unknown at this stage. Furthermore, it is important to acknowledge that the absolute differences between the two groups were relatively modest, although CB predilatation demonstrated statistically significant improvements in acute recoil and residual diameter stenosis compared with SB. Therefore, it remains uncertain whether these acute angiographic advantages will translate into meaningful long-term clinical benefits, such as late lumen enlargement or a reduced need for target lesion revascularization, whereas studies investigating the mechanisms of late lumen enlargement consistently indicate that the degree and depth of barotrauma are positive predictors of future vessel expansion [24–27]. Consequently, the true clinical relevance of our findings and the ultimate efficacy of CB in the DCB-only strategy will need to be confirmed through our ongoing 9-month angiographic, 6- and 1-year clinical follow-up analyses.

### Limitations of this trial

The NATURE trial is the first RCT to investigate the efficacy and safety of CB within a DCB-only strategy for de novo lesions using multimodality assessments, including angiography, IVUS, and FFR, in comparison with SB. This comprehensive dataset is instrumental in refining lesion preparation strategies and optimizing the selection of devices for DCB-only interventions.

However, this study has several limitations that should be acknowledged. First, this was an exploratory study, which may limit the statistical power to detect small but potentially meaningful differences between the groups. This study is particularly underpowered for the assessment of the primary endpoint with wide confidence intervals. Second, the open-label design, where operators were not blinded to the balloon type, could introduce potential bias in procedural conduct and decision-making, although the primary and key secondary endpoints were adjudicated by blinded core labs. Third, the study protocol mandated post-dilatation FFR measurement, which is not routinely performed in standard PCI practice. The unblinded nature of the intra-procedural FFR measurements introduces the potential for incorporation and procedural bias. According to the study protocol, FFR values obtained after lesion preparation were immediately available to the operators and were integrated into the multi-modality assessment to guide comprehensive decision-making, such as whether to proceed with a DCB-only strategy, perform additional lesion preparation, or transition to bailout stenting. Although the protocol did not pre-specify an exact FFR threshold for prompting additional procedures—instead allowing operators to utilize FFR as a continuous parameter of coronary function—an FFR > 0.80 was simultaneously defined as a core component of our primary composite endpoint. This dual role means that FFR functioned as both an outcome measure and a real-time treatment-guiding parameter. Consequently, knowledge of these FFR values inevitably influenced operator behaviour. For instance, operators might have accepted a suboptimal angiographic result (e.g., a residual stenosis > 30%) and proceeded with a DCB-only strategy if the concurrent FFR indicated an absence of significant flow limitation (FFR > 0.80), a pattern frequently observed in our study, particularly in the right coronary and left circumflex arteries (Figure S1). This inherent incorporation bias implies that the final procedural strategies and the achievement rates of the primary endpoint may not purely reflect the mechanical efficacy of the assigned predilatation balloons (cutting versus standard balloons), but rather a complex interaction between the physiological data and subsequent operator decisions. Fourth, the study protocol specified the use of the Wolverine™ CB and the Agent™ DCB. Therefore, the results may not be generalizable to other types of scoring/CBs or DCBs with different drug formulations and excipients. Finally, this report focuses on the acute procedural and 30-day outcomes. The long-term efficacy and safety, particularly the key secondary endpoint of late lumen loss at 9 months, will be crucial for a complete assessment of the two preparation strategies.

## Conclusion

The NATURE trial demonstrated that lesion preparation with CB was associated with greater acute luminal enlargement than SB in the setting of a DCB-only strategy. However, these angiographic advantages did not translate into a significantly higher rate of optimal lesion preparation according to the International DCB Consensus Group criteria. Further follow-up analyses and adequately powered studies are warranted to determine whether these acute benefits translate into improved long-term clinical and angiographic outcomes.

## Supplementary Information

Below is the link to the electronic supplementary material.

Supplementary file1 (DOCX 133 KB)

## Data Availability

The datasets generated and/or analyzed during the current study are not publicly available because they contain potentiallyidentifiable patient information but are available from the corresponding author upon reasonable request, subject to institutional review and applicable ethical and legal requirements.
