# Supplementary material for: Cutting vs standard balloon for lesion preparation in drug-coated balloon-only PCI: the NATURE randomised trial
Source: Cardiovasc Interv Ther. 2026 Aug 25;41(4):1053–67. doi: 10.1007/s12928-026-01330-x (PMC13582048; doi:10.1007/s12928-026-01330-x)
Supplement: Supplementary file 1 [file 12928_2026_1330_MOESM1_ESM.docx]

**Supplement**

**Cutting Balloon versus Standard Balloon for Lesion Preparation of Drug-Coated Balloon Treatment in De-novo Coronary Artery Lesions:**

**Primary Analysis of the NATURE Randomized Controlled Trial**

**Table S1. Inclusion and exclusion criteria of the NATURE trial**

| **1. Inclusion Criteria** | | |
| --- | --- | --- |
|  | 1) | Patients with chronic coronary syndrome or unstable angina without myocardial necrosis (no troponin elevation) who have a 90% stenotic lesion on CAG or indication of revascularization for confirmed myocardial ischemia. |
|  | 2) | Target lesion is a *de novo* coronary artery lesion with a reference vessel diameter of 2.5–4.0 mm and a lesion length of ≤28 mm, which is deemed suitable for DCB therapy. |
|  | 3) | Patients aged 18 years or older at the time of consent. |
|  | 4) | Patients who have signed the informed consent documents. |
|  | 5) | Patients who can be followed up for clinical events for 12 months post-PCI (including phone follow-ups). |
| **2. Exclusion Criteria** | | |
|  | **Patient exclusion criteria** | |
|  | 1) | Patients with acute myocardial infarction (irrespective of ST-segment elevation on ECG) or recent myocardial infarction. |
|  | 2) | Patients with unstable vital signs. |
|  | 3) | Patients with advanced coronary artery disease which is unsuitable for PCI (SYNTAX score ≥33). |
|  | 4) | Patients with impaired renal function (eGFR <30 mL/min), including those on dialysis. |
|  | 5) | Patients scheduled for surgeries or endoscopic procedures requiring discontinuation of antiplatelet therapy within 3 months post-PCI. |
|  | 6) | Patients scheduled for additional PCI within the study-target vessel. |
|  | 7) | Patients with allergic reactions to paclitaxel, everolimus, alloys (platinum-chromium, cobalt-chromium, stainless steel), contrast agents, metallic materials, or medications essential for PCI such as anti-platelet therapy, who cannot adequately receive prophylactic treatment. |
|  | 8) | Patients who are pregnant, breastfeeding, or potentially pregnant. |
|  | 9) | Patients planning to participate in or currently participating in other clinical trials or interventional clinical researches within 12 months after the procedure. |
|  | 10) | Patients deemed unsuitable for the study by the attending or primary physician. |
|  | **Lesion exclusion criteria** | |
|  | 1) | Lesions in the same vessel where PCI was performed within the past 12 months. |
|  | 2) | In-stent restenosis lesions including both 5 mm segments outside the stent edges. |
|  | 3) | Thrombotic lesions. |
|  | 4) | Lesions with dissection prior to PCI. |
|  | 5) | Chronic total occlusions. |
|  | 6) | Severely calcified lesions requiring or suitable for rotational or orbital atherectomy. |
|  | 7) | Lesions using directional coronary atherectomy (DCA). |
|  | 8) | Lesions using excimer laser coronary atherectomy. |
|  | 9) | Lesions in coronary artery bypass grafts (irrespective of arterial or venous). |
|  | 10) | Lesions protected by non-occluded coronary artery bypass grafts. |
|  | 11) | Lesions within 2 mm of the ostium of the right or left coronary artery. |
|  | 12) | Bifurcation lesions requiring stent implantation (including cases where stents will be implanted in other bifurcation segments). |
|  | 13) | Lesions deemed unsuitable for DCB or cutting balloon use. |

**Table S2. Participating Sites of the NATURE trial**

| **Medical Institution** | **Registered Cases** | **Representative investigators** |
| --- | --- | --- |
| Toho University Medical Center Ohashi Hospital | 34 | Raisuke Iijima,  Masato Nakamura |
| Kurashiki Central Hospital | 29 | Kohei Osakada  Kazushige Kadota |
| Kansai Rosai Hospital | 26 | Takayuki Ishihara |
| Chikamori Hospital | 24 | Koji Nishida |
| Kokura Memorial Hospital | 19 | Kenji Ando |
| Sakakibara Heart Institute | 15 | Mamoru Nanasato |
| Musashino Red Cross Hospital | 10 | Takashi Ashikaga |
| Teikyo University Hospital | 9 | Ken Kozuma |
| Mitsui Memorial Hospital | 9 | Kengo Tanabe |
| Fujita Health University Hospital | 8 | Takashi Muramatsu |
| Yokohama City University Hospital | 7 | Kiyoshi Hibi |
| Sakurabashi Watanabe Future Medical Center | 5 | Atsunori Okamura |
| Toyohashi Heart Center | 5 | Yoshihisa Kinoshita |
| Juntendo University Shizuoka Hospital | 4 | Satoru Suwa |
| Yokohama City University Medical Center | 2 | Kozo Okada |
| Miyazaki Medical Association Hospital | 2 | Nehiro Kuriyama |
| Kindai University Nara Hospital | 2 | Gaku Nakazawa |
| Kyoto University Hospital | 0 | Hiroki Shiomi |
| Sapporo Cardiovascular Clinic | 0 | Yutaka Tadano |

**Table S3. Primary Endpoint in PPS Analysis**

|  | **Cutting Balloon Group** | **Standard Balloon Group** | **Adjusted relative risk**  **(95% CI)** | **P value** |
| --- | --- | --- | --- | --- |
|  | N = 95 | N = 96 |  |  |
| 1. No flow-limiting dissection (TIMI flow grade 3) | 94 (98.9%) | 93 (96.9%) | 1.02  (0.98, 1.06) | 0.28 |
| 2. Residual DS ≤30% | 45 (47.4%) | 41 (42.7%) | 1.11  (0.84, 1.47) | 0.46 |
| 3. FFR >0.80 | 75 (78.9%) | 72 (75.0%) | 1.06  (0.90, 1.23) | 0.46 |
| 1, 2, 3. All criteria satisfied | 36 (37.9%) | 29 (30.2%) | 1.27  (0.86, 1.87) | 0.24 |

DS = diameter stenosis; FFR = fractional flow reserve; TIMI = Thrombolysis in Myocardial Infarction;

**Table S4. The post-predilatation angiographic, IVUS, and FFR findings between patients who successfully underwent a DCB-only strategy and those who required bailout stenting, stratified by the assigned treatment allocation (CB vs. SB).**

|  | **DCB-only strategy** | | **Bailout stenting** | |
| --- | --- | --- | --- | --- |
|  | Cutting  balloon N=80 | Standard balloon N=70 | Cutting  balloon N=21 | Standard balloon N=30 |
| Angiography | | | | |
| MLD, mm, (SD) | 1.67 (0.42) | 1.61 (0.36) | 1.60 (0.4) | 1.32 (0.4) |
| Residual DS, %, (SD) | 30.32 (11.56) | 31.06 (10.63) | 32.69 (12.67) | 42.36 (15.59) |
| Dissection |  |  |  |  |
| No dissection | 36 (45%) | 26 (37.1%) | 2 (9.5%) | 1 (3.3%) |
| A | 27 (33.8%) | 22 (31.4%) | 4 (19%) | 5 (16.7%) |
| B | 11 (13.8%) | 18 (25.7%) | 11 (52.4%) | 19 (63.3%) |
| C | 4 (5%) | 2 (2.9%) | 1 (4.8%) | 4 (13.3%) |
| E | 0 (0%) | 0 (0%) | 0 (0%) | 1 (3.3%) |
| Non-analyzable | 2 (2.5%) | 2 (2.9%) | 3 (14.3%) | 0 (0%) |
| IVUS | | | | |
| MLA | 3.71 (1.48) | 3.52 (1.43) | 3.26 (1.27) | 2.78 (1.15) |
| Minimum EEM area mm^2^, (SD) | 10.01 (4.11) | 9.88 (4.92) | 9.14 (3.64) | 8.42 (3.57) |
| Maximum plaque burden, %, (SD) | 70.50 (5.61) | 71.14 (6.41) | 71.73 (8.21) | 74.84 (6.35) |
| Area stenosis, %, (SD) | 51.17 (11.58) | 51.01 (11.99) | 56.25 (15.75) | 59.70 (13.34) |
| Dissection |  |  |  |  |
| No dissection | 27 (33.8%) | 17 (24.3%) | 4 (20.0%) | 9 (30.0%) |
| Intimal | 25 (31.3%) | 24 (34.3%) | 0 (0.0%) | 4 (13.3%) |
| Medial | 21 (26.3%) | 23 (32.9%) | 7 (35.0%) | 15 (50.0%) |
| Adventitial | 7 (8.8%) | 5 (7.1%) | 7 (35.0%) | 0 (0.0%) |
| Intramural hematpma | 0 (0.0%) | 1 (1.4%) | 2 (10.0%) | 2 (6.7%) |
| FFR | | | | |
| Post-predilatation FFR, (SD) | 0.88 (0.09) | 0.88 (0.08) | 0.82 (0.10) | 0.80 (0.09) |

DS = diameter stenosis; FFR = fractional flow reserve; EEM = external elastic membrane; IVUS = intravascular ultrasound; MLA = minimum lumen area; MLD = minimum lumen diameter

**Table S5. Operator’s Decision and Achievement of DCB-only Strategy in PPS Analysis**

|  | **Cutting Balloon Group** | **Standard Balloon Group** | **Adjusted relative risk** | **P value** |
| --- | --- | --- | --- | --- |
|  | N = 95 | N = 96 | **(95% CI)** |  |
| Operator’s Decision to Employ DCB-only Strategy after Lesion Preparation | 81 (85.3%) | 69 (71.9%) | 1.19  (1.02, 1.39) | 0.03 |
| Acievement of DCB-only Strategy without Bailout Stenting | 78 (82.1%) | 67 (69.8%) | 1.18  (1.00, 1.38) | 0.04 |

DCB = drug-coated balloon

**Figure S1. Correlation between post-predilatation FFR and post-predilatation %DS**

DS = diameter stenosis; FFR = fractional flow reserve; LAD = left anterior descending artery; LCX = left circumflex artery; RCA = right coronary artery.
